# Supplementary figures and images for: Equine alveolar macrophages and monocyte-derived macrophages respond differently to an inflammatory stimulus
Source: PLoS One. 2023 Mar 15;18(3):e0282738. doi: 10.1371/journal.pone.0282738 (PMC10016717; doi:10.1371/journal.pone.0282738)

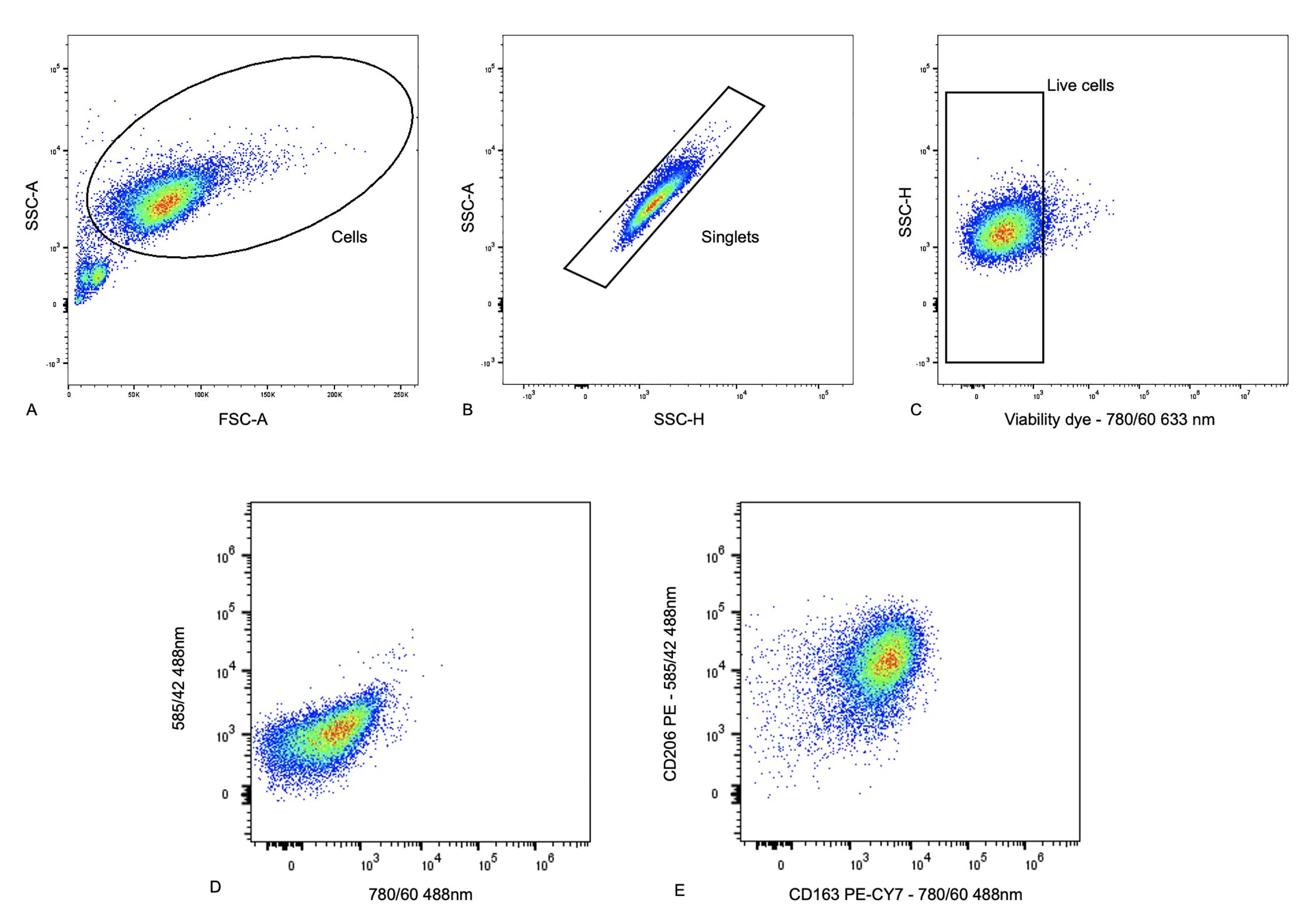

Supplement: S1 Fig — Monocyte-derived macrophages were uniform based on light scatter properties, thus a flow cytometry plot with their gating strategy is not provided. A: Cells were separated from debris based on size (forward scatter, FSC) and internal complexity (side scatter, SSC). B: Singlets, i.e., cells that passed through the laser beam one at a time were selected. C: Live cells were identified based on their negativity for the viability dye. D. Unstained control. E. Dual stained sample. The challenge mixture was composed of 106/mL Aspergillus fumigatus spores, 100 ng/mL LPS and 106/mL silica microspheres in 2 mL serum-free RPMI. (TIF) [file pone.0282738.s001.tif]
