# Supplementary material for: Equine alveolar macrophages and monocyte-derived macrophages respond differently to an inflammatory stimulus
Source: PLoS One. 2023 Mar 15;18(3):e0282738. doi: 10.1371/journal.pone.0282738 (PMC10016717; doi:10.1371/journal.pone.0282738)
Supplement: S1 Table — (DOCX) [file pone.0282738.s004.docx]

**S1 Table: Cellular composition of bronchoalveolar lavage fluid**

| Cell type | Horse 1 (%) | Horse 2  (%) | Horse 3  (%) | Horse 4  (%) | Horse 5  (%) | Horse 6  (%) |
| --- | --- | --- | --- | --- | --- | --- |
| Neutrophil | 2.2 | 2.6 | 1.0 | 3.8 | 4.0 | 1.2 |
| Lymphocyte | 37.8 | 24.4 | 44.2 | 39.4 | 49.0 | 28 |
| Macrophage | 58.8 | 70.8 | 53.8 | 56.0 | 46.4 | 70.4 |
| Mast cell | 1.2 | 2.0 | 1.0 | 0.8 | 0.6 | 0.4 |
| Eosinophil | 0 | 0.2 | 0 | 0 | 0 | 0 |
