# Supplementary material for: Equine alveolar macrophages and monocyte-derived macrophages respond differently to an inflammatory stimulus
Source: PLoS One. 2023 Mar 15;18(3):e0282738. doi: 10.1371/journal.pone.0282738 (PMC10016717; doi:10.1371/journal.pone.0282738)
Supplement: S2 Table — (DOCX) [file pone.0282738.s005.docx]

**S2 Table: P-values comparing cytokine production under different cell culture conditions**

| Cytokine | AMM^a^/C^b^ & AMM/T^c^ | MDM^d^/C & MDM/T | AMM/(T – C) & MDM/(T – C) |
| --- | --- | --- | --- |
| IL-1β^f^ | **0.0020** | n/a^e^ | n/a |
| IL-8^g^ | **0.0208** | **<.0001** | **<.0001** |
| IL-10^f^ | n/a | n/a | n/a |
| IL-12p70^f^ | 0.4331 | 0.2362 | 0.3493 |
| IFN-γ^f^ | n/a | n/a | n/a |
| TNF-α^f^ | **<.0001** | **<.0001** | **0.0382** |

^a^ Alveolar macrophage/monocytes

^b^ Control was exposure to serum free RPMI

^c^ Treatment was exposure to a mixture of fungal spores, LPS, and silica microspheres

^d^ Monocyte-derived macrophage

^e^ Not applicable. Data were not analyzed because at least one of the compared groups had cytokine concentrations below the limit of detection.

^f^ Data were not normally distributed, thus median concentrations were compared.

^g^ Data were normally distributed, thus mean concentrations were compared.
