# Supplementary material for: Equine alveolar macrophages and monocyte-derived macrophages respond differently to an inflammatory stimulus
Source: PLoS One. 2023 Mar 15;18(3):e0282738. doi: 10.1371/journal.pone.0282738 (PMC10016717; doi:10.1371/journal.pone.0282738)
Supplement: Attachment — Submitted filename: Response to Reviewers.docx [file pone.0282738.s007.docx]

ACADEMIC EDITOR: Although the authors have addressed most of the comments from the reviewers, there is one issue that needs to be fixed. I agree with the comments of reviewer-2 that the cytokine data that were "ND" or below the detection limit poses challenges in statistical calculations as presented in figure-2.  Therefore, I suggest the authors to plot individual data points (of those samples that were at/above the limit of detection) in Figure-2.

We have changed Figure 2 which now shows individual data points. Please see our responses to Reviewer 2 below.

Journal Requirements:

Please review your reference list to ensure that it is complete and correct. If you have cited papers that have been retracted, please include the rationale for doing so in the manuscript text, or remove these references and replace them with relevant current references. Any changes to the reference list should be mentioned in the rebuttal letter that accompanies your revised manuscript. If you need to cite a retracted article, indicate the article’s retracted status in the References list and also include a citation and full reference for the retraction notice.

No changes to the reference list were made because no retracted papers have been cited. The search was performed using this database: http://retractiondatabase.org/RetractionSearch.aspx#?auth%3dCavarra.

**Comments to the Author**

1. If the authors have adequately addressed your comments raised in a previous round of review and you feel that this manuscript is now acceptable for publication, you may indicate that here to bypass the “Comments to the Author” section, enter your conflict of interest statement in the “Confidential to Editor” section, and submit your "Accept" recommendation.

Reviewer #2: (No Response)

2. Is the manuscript technically sound, and do the data support the conclusions?

The manuscript must describe a technically sound piece of scientific research with data that supports the conclusions. Experiments must have been conducted rigorously, with appropriate controls, replication, and sample sizes. The conclusions must be drawn appropriately based on the data presented.

Reviewer #2: Partly

3. Has the statistical analysis been performed appropriately and rigorously?

Reviewer #2: No

4. Have the authors made all data underlying the findings in their manuscript fully available?

The [PLOS Data policy](http://www.plosone.org/static/policies.action#sharing) requires authors to make all data underlying the findings described in their manuscript fully available without restriction, with rare exception (please refer to the Data Availability Statement in the manuscript PDF file). The data should be provided as part of the manuscript or its supporting information, or deposited to a public repository. For example, in addition to summary statistics, the data points behind means, medians and variance measures should be available. If there are restrictions on publicly sharing data—e.g. participant privacy or use of data from a third party—those must be specified.

Reviewer #2: Yes

5. Is the manuscript presented in an intelligible fashion and written in standard English?

PLOS ONE does not copyedit accepted manuscripts, so the language in submitted articles must be clear, correct, and unambiguous. Any typographical or grammatical errors should be corrected at revision, so please note any specific errors here.

Reviewer #2: Yes

6. Review Comments to the Author

Please use the space provided to explain your answers to the questions above. You may also include additional comments for the author, including concerns about dual publication, research ethics, or publication ethics. (Please upload your review as an attachment if it exceeds 20,000 characters)

Reviewer #2: The authors addressed most of my concerns, with exception of the 2 below (1 new, minor).

1) The authors use throughout the paper the term cytokine multiplex measurement. Multiplex measurement just means multiple cytokines detected simultaneously, not the actual assay. Please consider using the term "bead-based multiplex assay" rather than multiplex measurement.

Thank you for the suggestion. This term was changed accordingly throughout the manuscript.

2) Authors: The flow cytometry figure was reformatted using dot plots to represent each horse. However, the cytokine figure cannot be presented in this way because many samples were “non-detects” therefore share the value 0. Instead, the cytokine figure was reformatted using mean/median and the confidence interval.

Thank you for the work on flow cytometry figures.

In the cytokines, if there were many “non-detects”, I believe the use of mean/median and confidence interval is not correct. In fact, in the Millipore Sigma website (link below) of the Milliplex MAP assay, one will find all sensitivities for the cytokines tested. When comparing to the raw data presented (thank you for sharing them), many values that are included in the analysis are actually below the sensitivity of the assay (essentially, also “non-detected” or “0”). In my opinion, the authors do not need statistics to demonstrate that there is stimulation of cytokines if they go from being undetected detected to a certain number (leave that to the Editor). For example, Figure 2, IL-1b. The limit of detection is 29.3 pg/ml. In the table with the raw data, if I am looking correctly, all controls except for 1 (30.83 pg/ml) are below the limit of detection (one could argue that 30.83 is too close to be considered valid). Similarly, in the treated group, if the authors remove the values below limit of detection (3.68 and 24.11), the median of the treated group will be higher. Please do this exercise for all cytokines, and add the individual values similar to Figure 1 (which now looks beautiful). I understand the frustration of having to report “non-detected” for many cytokines (for example, IL-12, INFg, and most of IL-8 data are below sensitivity). That is, however, part of reporting science and it may guide future research. An alternative approach is to concentrate the samples and try to measure again, but I understand that it may not be possible.

Thank you for the thoughtful comments, also for pointing out the sensitivity information with the link. We have changed Figure 2, which now shows individual values of each sample.

We agree that many of the concentrations were below the sensitivity of the assay and have removed some cytokines, for example IL-12 and IFN-g. However, we also note that the website gives a high cut-off point for each cytokine, which might lead us to omit important information. In addition, the limit of detection (LoD) given by the website is different from the LoD calculated based on the standard curve created by the Belysa® Immunoassay Curve Fitting Software (see link below). For IFNg, the standard curve gave an LoD of 14.96 pg/mL (cell F165 from the raw data file) whereas the website indicates the Lod of IFNg is 165 pg/mL. Therefore, we decided to keep some values which are just below the sensitivity concentration but still appear meaningful. For example, the LoD of IL-8 is 74.3 pg/mL according to the website, so we kept those samples with concentrations of IL-8 around 60 pg/mL, and removed those with extremely low values of ~ 10 pg/mL. We performed this exercise for all cytokines, and hope this is a rational justification that adequately addresses your concerns.
